# Hierarchical Y Zeolite-Based Catalysts for VGO Cracking: Impact of Carbonaceous Species on Catalyst Acidity and Specific Surface Area

**DOI:** 10.3390/molecules29133085

**Published:** 2024-06-28

**Authors:** Jayson Fals, Juan Francisco Garcia-Valencia, Esneyder Puello-Polo, Fernando Tuler, Edgar Márquez

**Affiliations:** 1 Grupo de Investigación en Oxi/Hidrotratamiento Catalítico y Nuevos Materiales, Programa de Química-Ciencias Básicas, Universidad del Atlántico, Barranquilla 080003, Colombia; jgarciavalencia@mail.uniatlantico.edu.co (J.F.G.-V.); esneyderpuello@mail.uniatlantico.edu.co (E.P.-P.); 2 Instituto de Investigaciones en Catálisis y Petroquímica, INCAPE (FIQ, UNL, CONICET), Centro Científico Tecnológico CONICET Santa Fe, Colectora Ruta Nac. No. 168, Km. 0, Paraje El Pozo, Santa Fe 3000, Argentina; ftuler@fiq.unl.edu.ar; 3 Grupo de Investigación en Química y Biología, Departamento de Química y Biología, Facultad de Ciencias Básicas, Universidad del Norte, Barranquilla 080003, Colombia

**Keywords:** zeolite, catalyst, VGO cracking, acidity, surface area

## Abstract

The performance of catalysts prepared from hierarchical Y zeolites has been studied during the conversion of vacuum gas oil (VGO) into higher-value products. Two different catalysts have been studied: CatY.0.00 was obtained from the standard zeolite (Y-0.00-M: without alkaline treatment) and CatY.0.20 was prepared from the desilicated zeolite (Y-0-20-M: treated with 0.20 M NaOH). The cracking tests were carried out in a microactivity test (MAT) unit with a fixed-bed reactor at 550 °C in the 20–50 s reaction time range, with a catalyst mass of 3 g and a mass flow rate of VGO of 2.0 g/min. The products obtained were grouped according to their boiling point range in dry gas (DG), liquefied petroleum gas (LPG), naphtha, and coke. The results showed a greater conversion and selectivity to gasoline with the CatY.0.20 catalyst, along with improved quality (RON) of the C_5_–C_12_ cut. Conversely, the CatY.0.00 catalyst (obtained from the Y-0.00-M zeolite) showed greater selectivity to gases (DG and LPG), attributable to the electronic confinement effect within the microporous channels of the zeolite. The nature of coke has been studied using different analysis techniques and the impact on the catalysts by comparing the properties of the fresh and deactivated catalysts. The coke deposited on the catalyst surfaces was responsible for the loss of activity; however, the CatY.0.20 catalyst showed greater resistance to deactivation by coke, despite showing the highest selectivity. Given that the reaction occurs in the acid sites of the zeolite and not in the matrix, the increased degree of mesoporosity of the zeolite in the CatY.0.20 catalyst facilitated the outward diffusion of products from the zeolitic channels to the matrix, thereby preserving greater activity.

## 1. Introduction

Crude oil is a non-renewable resource and serves as the primary source of energy and raw materials for generating a diverse array of products, including liquid fuels such as gasoline and diesel, as well as olefins, among others [1,2,3]. Typically comprised of complex mixtures of hydrocarbons with high molecular weights and boiling points spanning a wide range, crude oil requires processing with catalysts in refineries to yield products of higher added value [4]. Among the conversion processes in refineries, fluidized bed catalytic cracking of hydrocarbons (FCC) is widely regarded as the most profitable and versatile. These FCC units are adept at processing heavy feedstock, with their typical feed being vacuum gas oil (VGO) obtained from the distillation of crude oil under vacuum conditions [5,6]. Different hydrocarbon fractions can be distinguished from VGO according to the degree of polarity, and four fractions can be separated (SARA): saturated, aromatic, resins, and asphaltenes [7]. The aromatic and resin fractions are the most voluminous (largest kinetic diameter), thus making their diffusion into the microporous channels of the zeolites difficult (pore size = 7.4 Å) [8]. In commercial catalysts, zeolites typically constitute between 20 and 40 wt%, supported on a matrix, which can be either active or inactive. Among their crucial properties are acidity (including concentration, nature, and strength) as well as textural characteristics, such as BET specific surface area and pore volume, among others [9]. These properties are mainly responsible for the activity and selectivity of these catalysts.

The microporous nature of zeolites renders them selective for molecules with diameters smaller than their pore size [9,10]. This restriction in the diffusion of bulky molecules impacts their activity, as these molecules may not diffuse toward the acidic sites of the catalyst. The diffusion restrictions in Y zeolite micropores could be decreased following different routes. The first one consists of synthesizing zeolites with extra-large pores, however, it is very expensive and has low hydrothermal stability. The second one is based on the use of a smaller Y zeolite crystal size so as to increase the external specific surface area and decrease diffusion paths in the crystals. The third (alkaline leaching) and most profitable is the induction of intracrystalline mesopores, that is, cracks, cavities, and channels in the crystals in the mesopore size range [11]. Leaching in an alkaline medium stands out as one of the simplest and most efficient techniques for selectively removing silicon atoms from the crystalline lattice, thereby inducing the partial destruction of the network, leading to interconnected mesopores [12]. The treatment consists of the removal of silicon atoms from the zeolite crystalline network, thus inducing the partial destruction of the network and consequently leading to the creation of a hierarchical structure as mesopore formation occurs. If conditions are appropriate, it is possible to attain a catalyst with the desired activity and selectivity in the micropores and intracrystalline mesopores helping reactants, intermediates, and products of the reaction to diffuse more easily, thereby enhancing the achieved conversion and mitigating deactivation resulting from coke deposition [13]. In this sense, Groen et al. [14] demonstrated that the diffusion time of neopentane in desilicated zeolites could be reduced by up to two orders of magnitude in relation to untreated microporous zeolites, concluding that an interconnected mesoporous system was formed.

Given the carbocationic mechanisms governing the complex set of reactions in the cracking over zeolite Y, the performance of a given catalyst is strongly constrained by its acidic properties. Then, both the activity and selectivity toward different products will depend on the density, nature (Brönsted or Lewis), and strength of the acid sites. In addition to acidity, the catalyst’s textural properties also play an important role in the observed activity and selectivity, as well as in catalyst deactivation. The rapid deactivation as cracking reactions proceed results in the formation of carbonaceous deposits (coke). The diverse array of chemical reactions leading to coke formation significantly impacts activity, as it deposits on active acid sites and blocks pores. The amount of coke produced and its nature (H/C ratio and degree of condensation) will depend on the composition of the feedstock, catalyst properties, and the process conditions [15,16,17].

Hydrocarbon-cracking catalysts need to undergo evaluation on a small scale in laboratory reactors before being deployed in industrial processes. The advantages of utilizing these laboratory reactors include lower costs and shorter study durations, enabling the requesting company to obtain results that favor the economics of the refining process [18]. In this regard, various methodologies have been developed for this purpose, encompassing different reactor configurations and operating conditions. One such method is the micro activity test (MAT), as defined by the ASTM D-3907/03 standard. The MAT aims to replicate the continuous fluidized bed riser reactor by utilizing a fixed-continuous-bed reactor. Its advantages include simplicity, low manufacturing and operational costs, and the potential for automation. Although the fluid dynamics may not perfectly align with the industrial process, the conversion and selectivity data obtained from the MAT are comparable to plant data [18,19,20].

In the intricate landscape of refinery operations, catalysts play a pivotal role. These meticulously formulated agents are tailored to the specific attributes of the feedstock they process. Our study focuses on a critical aspect: the catalytic efficacy of two distinct zeolitic catalysts synthesized from hierarchical zeolites. These catalysts serve as gatekeepers, facilitating the diffusion of reactant molecules toward active sites while expediting the outward flow of products through zeolitic channels. Moreover, we delve into the nature of carbonaceous residues deposited on these catalysts, unraveling their impact on both acidity and structural properties. In this investigation of these zeolitic catalysts, we not only elucidate their intricate roles, but also pave the way for refining and optimizing critical refinery processes.

## 2. Results and Discussion

### 2.1. Zeolites and Catalyst Properties

The textural and crystalline properties of the hydrothermally stabilized zeolites and the catalysts prepared from them were studied and the results are shown in Table 1. As expected, the alkaline treatment of the standard zeolite increased its mesoporosity, as indicated by the greater surface area, volume, and average diameter of mesopores. This enhancement is expected to improve the diffusion of bulky molecules toward the active sites of the zeolite [21]. The removal of silicon from the crystal lattice resulted in an excessive loss of crystallinity, evidenced by a decrease in unit cell values. Therefore, the specific surface area was also strongly affected by the destruction of zeolite crystals caused by the desilication treatment. Similar results were reported by García et al. [22] after treating Y zeolites with NaOH at different concentrations. They observed an increase in mesoporosity with the alkaline treatment, accompanied by a loss of crystallinity of up to 46%.

The prepared catalysts exhibited a similar trend to the original zeolites, with CatY.0.20 displaying the highest mesoporosity. In this way, the mesopore area followed the trend: Y-0.00-M < Y-0.20-M and CatY.0.00 < CatY0.20. The mesopore volume followed the same trend, increasing with the severity of desilication. In the zeolites, the mesopore volume expanded from 0.299 to 0.677 cm^3^ g^−1^, while in the catalysts, it ranged from 0.551 to 0.669 cm^3^ g^−1^. Furthermore, since the average mesopore diameter was strongly related to the mesopore area, an increase in the latter entailed an increase in the former. In this regard, CatY.0.20 exhibited the largest average mesopore diameter, measuring 99.7 Å. This underscores the importance of selecting suitable zeolites for formulating catalysts tailored to specific feedstock processing requirements. On the other hand, XRD analysis revealed that the alkaline treatment reduced the crystallinity of the material, a behavior associated with the selective removal of Si from the zeolite structure [23]. These changes in physical properties with desilication treatment are in line with those previously reported by other authors for different zeolites [24,25]. Consequently, it is expected that CatY.0.20 would facilitate the diffusion of bulky molecules characteristic of the aromatic fractions and resins present in VGO.

Table 2 shows the distribution of acid sites (nature and strength) in the hydrothermally stabilized zeolites and the composite catalysts. After the alkaline treatment, an increase in total acidity was observed (both in Brönsted and Lewis sites), represented by the amount of pyridine that remained absorbed after desorption at 150 °C; this increase was more significant in the Lewis sites. Higher concentrations of Lewis sites occurred as a consequence of the deposition of extra-lattice aluminum during desilication, which was reflected in the decrease in the ratio of B/L acid sites. However, at the same time as the deposition of extra-red aluminum occurred, the removal of silicon also occurred, giving rise to new Brönsted sites. Sadowska et al. observed a similar trend in acidity when treating ZSM-5 zeolites with NaOH solutions of different concentrations (up to 1.0 M). Specifically, for samples treated between 0.1 and 0.5 M NaOH, the density of both Brönsted and Lewis acid sites increased compared with the starting zeolite [26].

As expected, the total acidity values of both catalysts decreased significantly as a result of the zeolite dilution in an inert matrix. CatY.0.00 was the most affected, experiencing losses of up to 70%, while CatY.0.20 reached a maximum reduction of 65%. The strongest Brönsted acid sites, represented in Table 2 by the desorption temperatures of 300 °C and 450 °C, primarily facilitated cracking reactions. In this regard, the CatY.0.20 catalyst showed a higher abundance of Brönsted acid sites with medium and strong acidic strength, with a total concentration of 33 μmol Py/g compared with 25 μmol Py/g for CatY.0.00. In principle, this acidic property, coupled with its elevated mesoporosity (refer to Table 1), could potentially enhance the conversion of heavier feeds. These characterization results of zeolites and catalysts derived from zeolites underscore the significance of modifying zeolites through desilication, assessing them in a laboratory reactor, and subsequently formulating catalysts tailored to the specific demands of the process.

### 2.2. Catalytic Performance

#### Conversion and Product Distribution

**Figure 1:**
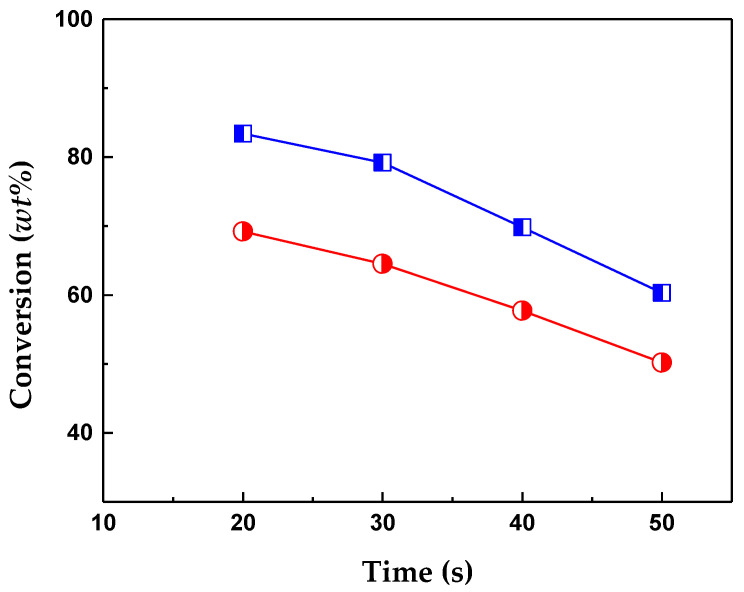
VGO conversion as a function of reaction time at 550 °C on CatY.0.00 (●) and CatY.0.20 (□).

The catalytic performance of the two catalysts prepared from hierarchical zeolites was evaluated in the conversion of VGO vacuum gas oil from a Colombian refinery. The temperature (550 °C) and reaction times (ranging between 20 and 50 s) employed in this study are representative of those commonly utilized in commercial fluidized bed catalytic cracking processes for fluid catalytic cracking (FCC). The experiments were conducted in a MAT type reactor, which, along with the CREC riser reactor, is widely employed for evaluating catalytic cracking catalysts. Figure 1 shows the conversion of VGO over both catalysts at 550 °C. As can be seen, both textural and acidic properties influenced conversion. The observed conversion profiles decreased due to the continuous contact of the fresh feed with a fixed catalyst bed, which experienced deactivation from coke deposits and underwent changes in activity profiles over time. Consequently, conversion decreased as residence time increased [18].

The CatY.0.20 catalyst, with the highest mesoporosity and amount of Brönsted acid sites, showed the highest conversion, with maximum values of up to 83.4 wt%. The increased intracrystalline mesoporosity favored the diffusion transport of the bulky molecules present in the VGO, thus improving the access to the active sites for cracking and therefore presenting the highest conversions. At longer reaction times, the conversion decreased, obtaining the lowest conversions of 60.3 wt% for CatY.0.20 and 50.2 wt% for CatY.0.00. The conversion efficiencies observed in this study are comparable to those reported for commercial systems, which typically exhibit values ranging from 60 wt% to 90 wt%. These variations are contingent upon the power supply specifications and operational parameters [15].

The distributions of the products and hydrocarbons in the gasoline boiling range in the conversion of VGO are shown in Table 3 in the form of selectivities. Differences in the properties of catalysts have impacted the yield of gasoline and, notably, gas production. At the industrial scale, existing refineries are currently oriented toward the production of liquid fuels, such as gasoline, with selectivities exceeding 40 wt% [27]. Consequently, the CatY.0.20 catalyst, characterized by the highest degree of mesoporosity and acidity, exhibited the highest selectivities toward the gasoline cut, ranging between 42 wt% and 51 wt%, while the CatY.0.00 catalyst reached maximum selectivities of 35 wt%. This difference arose due to the greater microporosity of the CatY0.00 catalyst, which hindered the cracking mechanism of VGO hydrocarbon compounds. On the other hand, the greater mesoporosity in CatY0.20 improved the diffusion of the primary product gasoline, thus reducing the possibility of overcracking to lighter products. In this mechanism, alkanes in the gasoline fraction dehydrogenate to form alkenes at the metal sites. This is where the hydrogenation/dehydrogenation capability of the metallic phase becomes crucial. Subsequently, the desorption of the formed alkenes from the metal site takes place, followed by their migration to the Brönsted acid sites. At these sites, the alkenes undergo protonation to form secondary alkylcarbenium ions [28].

In relation to the composition of the gasoline cut, the fraction derived from CatY.0.20 exhibited the highest aromatic selectivity (46.3 wt%), in comparison with the catalyst CatY.0.00 (34.7 wt%). To comprehensively characterize the gasoline fraction, the RON of this fraction was ascertained, adhering to the methodology delineated by Anderson et al. [29]. In this manner, it could be observed that the research octane number demonstrated an increasing trend with the increase in the mesoporosity degree of the catalyst. This increase was a direct consequence of the increase in the concentration of aromatics in the gasoline fraction, as observed for CatY.0.20. The values obtained for the catalyst possessing the highest mesoporosity were notably high (88.2) compared with commercially obtained values [30]. On the other hand, compared with CatY.0.00, the production of paraffinic gasoline was favored, resulting in a lower quality of the gasoline cut, with maximum values reaching 81.0.

Regarding the gas cut, the catalyst with a higher degree of microporosity, CatY.0.00, favored overcracking, resulting in an increased yield of gases with LPG selectivities of up to 34 wt% and DG of 29 wt%. Although CatY.0.00 had a lower concentration of acid sites compared with the CatY.0.20 catalyst, this behavior could be attributed to an electronic confinement effect within the microporous crystals of the zeolite, enhancing the interaction of reactant molecules with acid sites and consequently promoting the overcracking of hydrocarbons, primarily of the paraffinic type [31]. As expected, the gas fraction comprised solely paraffins. Specifically, methane and ethane are recognized as dry gas (DG), while propane, isobutane, and n-butane are typically categorized as liquefied petroleum gases (LPG). The greater selectivity of LPG observed in the gaseous products over both catalysts to DG is explained by the overcracking that converts molecules in the gasoline range (C_5_–C_12_) into liquefied petroleum gas. However, catalysts and zeolites with low acidity exhibit greater thermal cracking activity, leading to the production of low-molecular-weight gaseous products (C_1_ and C_2_) through free radical mechanisms. This behavior of increasing relative relevance of thermal cracking when the extent of catalytic cracking decreases is described in the literature [32,33]. For example, García et al. [34] reported a detailed analysis of the catalytic cracking of bio-oils improved by the formation of mesopores by means of Y zeolite desilication, and a similar trend was observed in the distribution and quality of the products obtained.

The programmed temperature oxidation technique allowed us to quantify the amount of coke deposited on the surface of the catalyst and to know its nature based on the interaction between the acid sites of the catalyst and the basic coke molecules [35]. The coke yield was also slightly favored by the widening of the porous structure, and the highest values were obtained with CatY.0.20 with selectivities of up to 19 wt%. However, despite exhibiting a higher selectivity toward coke formation, the catalyst with greater mesoporosity demonstrated greater resistance to deactivation, thereby retaining higher activity compared to the CatY.0.00 catalyst. This behavior was attributed to the larger average pore diameter present in the catalyst with greater mesoporosity, which facilitated the diffusion of products outward from the zeolitic channels. Consequently, this prevented pore blockage by carbonaceous deposits, with pore blockage being the primary cause of activity loss in zeolitic catalysts [36]. Similar results were observed in various reactions by our research group, wherein the desilication treatment of Y zeolites enhanced resistance to deactivation by coke [8].

**Figure 2:**
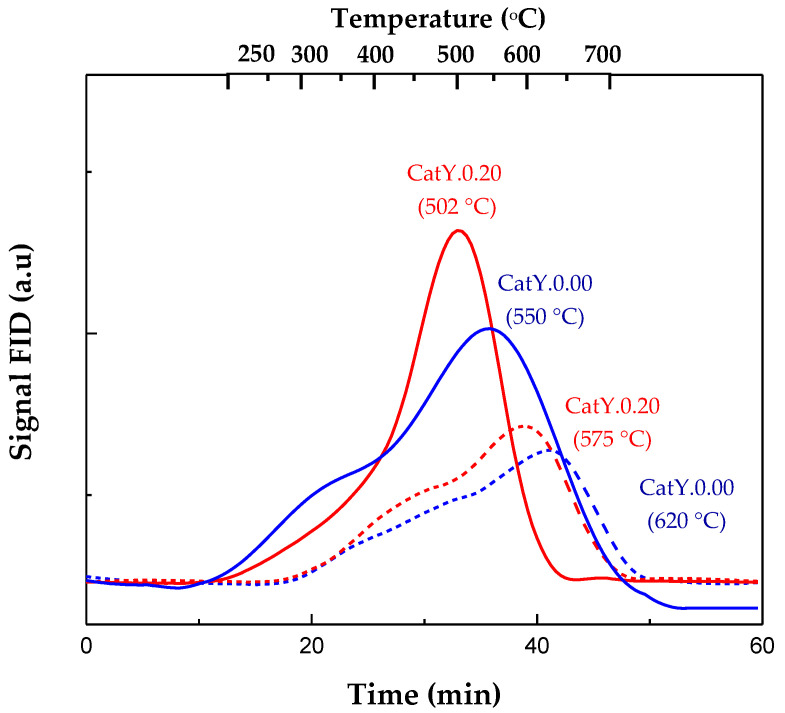
Combustion profiles (TPO) of the coke formed in the cracking of VGO on CatY.0.00 and CatY.0.20. Maximum (dashed line) and minimum conversion (solid line).

The TPO profiles for the two coked catalysts (CatY.0.00 y CatY.0.20) are plotted in Figure 2; the solid line represents the maximum conversion achieved, while the dashed line represents the minimum conversion. These profiles can be divided into two peaks, as reported in the literature [37]. The first peak occurs within a low-temperature range (200–450 °C) and is associated with a fraction of coke deposited on the external surface of zeolite crystals. The combustion of this fraction is not limited by diffusion constraints. This coke typically exhibits a poorly developed structure with a high H/C ratio. In contrast, the primary peak observed at higher temperatures (450–650 °C) corresponds to the fraction of coke located inside the crystalline channels of the zeolite. The combustion of this fraction is hindered by diffusional limitations [38]. These diffusional constraints in the catalyst with greater microporosity (CatY.0.00) prolong the residence time of molecules within the internal channels of the zeolite. This extended residence time enhances their propensity to condense, ultimately leading to the formation of polyaromatic structures that require higher temperatures for combustion [39]. On the contrary, the higher mesopore volume in catalyst CatY-0.20, allows for the diffusion of a higher proportion of aromatic molecules present in coke as compared with catalyst CatY-0.00, thus resulting in a less-condensed coke that combusts at lower temperatures. Furthermore, the peaks shifting to higher temperatures also suggest a strong interaction between the acidic sites of the zeolite and the basic sites of the carbonaceous deposits. In this regard, the CatY.0.00 catalyst, with the highest concentration of strong acid sites (Tdesorption > 450 °C), exhibited the most significant shifts in its coke combustion peaks. At minimum conversion (solid line), it was observed in the CatY.0.00 catalyst that there was a broadening of the TPO profile during the combustion of the deposited coke. This broadening would indicate that the nature of these carbonaceous deposits was more heterogeneous compared with those deposited on CatY.0.20. These results are consistent with those previously reported by other authors [36,40,41,42].

**Figure 3:**
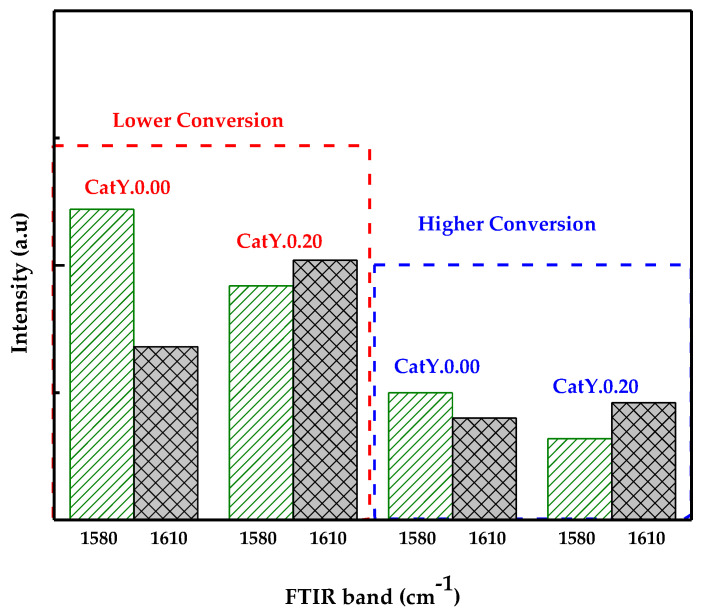
Intensities of the FTIR bands assigned to aromatic coke (1580 cm^−1^) and olefinic coke (1610 cm^−1^) formed in the conversion of VGO at 550 °C.

Continuing the investigation into the nature of coke, the results obtained from the TPO profiles are complemented by the programmed temperature desorption (Py-TPD) technique using pyridine as a probe molecule and infrared spectroscopy (FTIR). Through this analysis, two types of coke can be identified, the FTIR band assigned to aromatic coke (1580 cm^−1^) and that assigned to olefinic coke (1610 cm^−1^). Figure 3 shows the intensities of the bands at 1580 and 1610 cm^−1^ observed on the catalysts after cracking VGO. These observed results are consistent with the combustion profiles shown in Figure 2, where the combustion peaks of the CatY.0.20 catalysts were observed to shift to lower temperatures. Additionally, a greater amplitude in the combustion peaks indicated the presence of heterogeneous coke. In this regard, CatY.0.00 exhibited predominantly aromatic coke, particularly at low conversions, where the amount of olefinic coke doubled. These results align with findings reported by other authors, such as Bauer and Karge [43]. They characterized coke deposited on various zeolites and observed a greater presence of aromatic coke in zeolites with a higher degree of microporosity [44]. In general, both types of coke (aromatic and olefinic) were obtained; however, the olefinic nature prevailed in CatY.0.20. Another important aspect is that, at lower conversions (higher coke production), the highest intensities of both types of coke were obtained.

**Figure 4:**
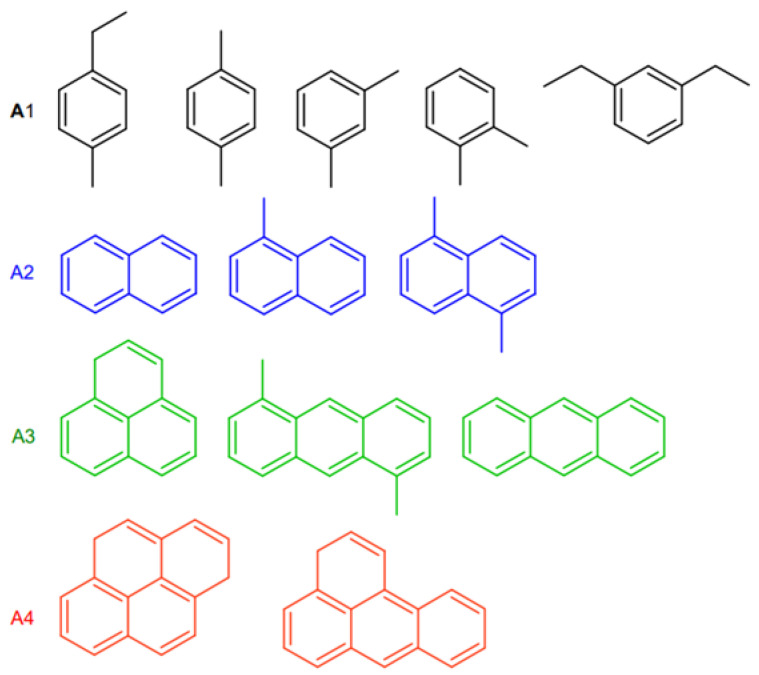
Content of soluble coke deposited on the spent catalysts used in the cracking of VGO.

Figure 4 shows the main compounds of the soluble coke fraction in the coked catalysts. The coke compounds are predominantly represented by aromatic species with one, two, three, and four rings, denoted as A1, A2, A3, and A4, respectively. It was noted that the catalyst with the highest degree of microporosity (CatY.0.00) exhibited a significant presence of aromatic species with a higher degree of condensation (70% by weight of A4). The results are consistent with those shown in Figure 3 where CatY.0.00 presented a higher proportion of aromatic coke. In contrast, the catalyst CatY.0.20 with higher macroporosity showed the presence of species with a lower degree of condensation (70% by weight of A1 + A2 + A3). It is believed that a portion of the coke was trapped within the microporous structure of the coked catalyst, while the remainder consisted of polyaromatic species deposited within the matrix (comprising meso and macropores).

**Figure 5:**
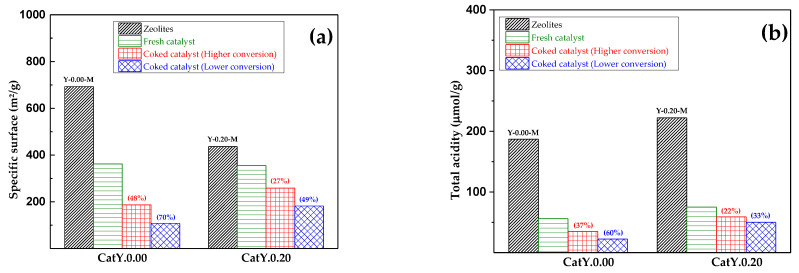
Deterioration of the specific BET surface area (**a**) and the total acidity (**b**) of coked catalysts.

The deterioration of the textural and acidic properties of the catalysts is illustrated in Figure 5. Figure 5a depicts the decrease in the BET specific surface area, whereas Figure 5b displays the decrease in the total acidity during the reaction. The catalyst with the highest mesoporosity (CatY.0.20) showed greater resistance to the negative effects caused by coke deposition, showing maximum specific surface area losses of 49%, while CatY.0.00 was the most affected, with losses of up to 70%. This behavior could be explained by the mesopores generated in the zeolite Y of the CatY.0.20 catalyst, which allowed the diffusion of the aromatic coke precursor molecules out of the zeolite channels, thus preventing their growth and subsequent pore blocking. This same behavior was previously observed by other authors [44,45,46]. Regarding total acidity, observably, the catalyst with the highest microporosity experienced the most significant decline in acidity, with losses reaching up to 60%, whereas CatY.0.20 exhibited losses of only up to 33%, thereby retaining a substantial percentage of its activity for subsequent cracking cycles. Considering the cyclical nature of cracking processes, wherein the catalyst underwent alternation between reaction and regeneration units, it became imperative to investigate the catalyst’s evolution across multiple reaction/regeneration cycles.

## 3. Materials and Methods

### 3.1. Alkaline Treatment and Hydrothermal Stabilization of Y Zeolites

A commercial Y zeolite (CBV-760, Si/Al = 31) was supplied from Zeolyst International and underwent a leaching process in an alkaline medium (desilication), as previously described by our research group [10]. Subsequently, both the untreated and desilicated zeolites were subjected to hydrothermal stabilization for 4 h at a temperature of 750 °C in the presence of steam. For desilication, 4 g of commercial zeolite Y was suspended in 100 mL of NaOH solution (0.20 M) and stirred continuously for 15 min at room temperature. The zeolite was subsequently treated with an HCl solution (1.00 M) until reaching a final pH of 2.00. The resulting solution was then separated by filtration and subjected to three exchange cycles using an NH_4_Cl solution (0.50 M). The product obtained was washed with deionized water and filtered under vacuum conditions. Finally, both the base and modified zeolite were hydrothermally stabilized and named Y-0.00-M and Y-0.20-M, respectively. The hydrothermal treatment was carried out in a steel tubular reactor. In a typical experiment, 2 g of zeolite was placed in the reactor and then introduced into a cylindrical furnace. The zeolite was heated with a rate of 5 °C/min until it reached a temperature of 250 °C. Once the temperature stabilized for 5 min, water was supplied to the reactor at a constant flow rate close to 1.0 g/min. The heating process continued at 10 °C/min until it reached a temperature of 780 °C. To determine the performance, the ratio of the mass of zeolite recovered after hydrothermal treatment to the initial mass loaded into the reactor was calculated, yielding values greater than 96 wt%.

### 3.2. Catalyst Synthesis

To adequately evaluate the catalytic performance of the hierarchical zeolite in a MAT type fixed-bed laboratory reactor, the zeolite was used in the synthesis of a composite catalyst with a typical FCC catalyst formulation [37]. In this study, the FCC catalysts were prepared following the typical formulation used in commercial FCC catalysts, comprising 35 wt% zeolite Y, 45 wt% matrix, and 20 wt% ligand. As a support, an inert Merck chromatography-grade mesoporous silica matrix was utilized. This matrix was pre-sieved within the range of 75–105 μm and calcined for 2 h at 550 °C with a heating rate of 12 °C/min. Then, 3 g of zeolite Y calcined at 550 °C was thoroughly mixed with 5 g of previously sieved and calcined silica matrix. Separately, 5 g of 40 wt% colloidal SiO_2_ (Ludox AS-40) was mixed with 5 g of deionized water to form a homogeneous paste. This paste was dried in an oven at 110 °C for 12 h, and the resulting solid was crushed and sieved within the range of 75–105 μm. The sieved powder was calcined in a muffle furnace with a rate of 12 °C/min up to 550 °C and held at that constant temperature for 4 h. Finally, the composite catalysts were named CatY-0.00 for the catalyst obtained from zeolite Y-0.00-M and CatY-0.20 for the catalyst obtained from zeolite Y-0.20-M. The yield was determined by calculating the ratio of the mass of the calcined catalyst to the sum of the masses (on a dry basis) of the various components used in the formulation. This yield varied within the range of 85–95 wt%.

### 3.3. Physicochemical Characterization of Zeolites and Catalysts

The zeolites, both standard and modified, as well as the composite catalysts prepared from the hierarchical zeolites, underwent characterization using various analytical techniques.

X-ray diffraction (XRD) was performed to study the crystalline properties and determine the unit cell size based on ASTM D-3906 [47] and ASTM D-3942 [48] standards, respectively. The analysis was performed using a Siemens D500 X-ray diffractometer with a monochromatic CuKα radiation source (λ = 1.5418 Å). Data were obtained in the 2θ range of 5–50° with a step size of 0.05° and a dwell time of 3 s per step.

The textural properties of the zeolites were assessed using nitrogen adsorption at −196 °C using a micromeritics automatic analyzer. Before analysis, the samples were degassed at 300 °C for 24 h under vacuum conditions of 10^−6^ mmHg. The Brunauer–Emmet–Teller model (BET) was used in the 0.005 < P/Po < 0.030 range to determine the specific surface area. The total pore volume was assessed using the amount of nitrogen adsorbed until P/Po~0.98. The micropore volume and the mesopore-specific surface area were estimated with the t-plot method. The Barrett–Joyner–Halenda model (BJH) was used in order to determine the average mesopore diameter.

Inductively coupled plasma–optical emission spectroscopy (ICP–OES) was employed to determine the concentrations of Si, Al, and Na in the samples. The samples were powdered, dried for 2 h at 110 °C, weighed, and then digested with hydrofluoric acid using a Milestone START D microwave digester. Finally, the solution obtained was filtered and diluted to a known volume with deionized water, to subsequently be analyzed in a Perkin Elmer model OPTIMA 7300 DV spectrometer.

Pyridine adsorption infrared spectrometry (Py—FTIR) was performed to determine the nature and strength of the acid sites present in fresh and coked zeolites and catalysts. Tablets with a diameter of 15 mm were prepared using 80 mg of the solid at a pressure of 4 tons/cm^2^. The samples were degassed for 2 h at 450 °C and 10^−3^ Torr and then the spectrum was obtained after cooling to room temperature. The pyridine molecules were adsorbed at 100 °C, while their desorption was carried out at 150, 300, and 400 °C. Spectra were obtained at a resolution of 4 cm^−1^ and 256 scans with a Nicolet FTIR spectrophotometer. The FTIR absorption bands at 1540 and 1450 cm^−1^ were identified as the Brönsted and Lewis acid sites due to the formation of pyridinium and pyridine ions, respectively. The background spectra obtained for the coked zeolites were also used to elucidate the nature of the carbonaceous deposits. The signal at 1580 cm^−1^ was assigned to coke of an aromatic nature and the signal at 1610 cm^−1^ was assigned to olefinic coke, as previously reported [49].

Temperature-programmed oxidation (TPO) was performed to study the nature and quantify the deposited coke. The carbonaceous deposits were oxidized in a stream of oxygen diluted in nitrogen (1% *v*/*v*). Carbon monoxide (CO) and carbon dioxide (CO_2_) were generated as combustion products, which were subsequently converted into methane using a nickel catalyst in the presence of hydrogen. The methane produced was quantified using a flame ionization detector (FID). Mass balances were greater than 95 wt% in all cases. Additionally, the coke trapped in the porous structure of the catalyst was removed by treatment with hydrofluoric acid. Then, by liquid–solid extraction with dichloromethane, soluble coke was obtained and analyzed by gas chromatography. The soluble structures were the lightest fractions of coke and contained most of the compounds trapped in the micropores of the zeolite.

### 3.4. VGO Characterization

Given the importance of adequate characterization of the feedstock, multiple analysis techniques were employed, as outlined below: API gravity was determined using the ASTM D287-12b standard [50]. The Conradson-type carbon residue (CCR) was obtained through a calcination method using the ASTM D4530-15 standard [51]. Simulated distillation curves were generated using a Shimadzu GC-2014 gas chromatograph, following the ASTM D1160 procedure [52]. The saturated, aromatic, resin, and asphaltene (SARA) fractions were isolated from the VGOs following the procedure outlined in ASTM D2007–11 [53]. Elemental analysis, including nickel, vanadium, sodium, iron, and copper content, was conducted using inductively coupled plasma–optical emission spectroscopy (ICP-OES) with a PerkinElmer model OPTIMA 7300 DV spectrometer. The most important properties of the feedstock used are presented in Table 4.

#### SARA VGO Fractioning

SARA fractionation was conducted in two stages. Initially, the asphaltenes (AsFs) were isolated by precipitation with n-pentane (Merck, >99%). Subsequently, the remaining fractions (SF, AF, and RF, referred to as SAR) were separated via column chromatography, employing a sequence of organic solvents arranged by increasing polarity. All fractions were concentrated using a rotary evaporator at 38 °C and 415 Torr. The complete removal of solvent from each fraction after treatment was confirmed through gas chromatography analysis. The concentrations of the various hydrocarbon groups in the VGO are also outlined in Table 4.

## 4. Conclusions

Alkaline treatment of zeolite Y improved the diffusion of reactants and products within the porous structure of the zeolite. This treatment not only increased conversion values, but also improved resistance to deactivation by coke. Additionally, the results obtained from the cracking of Colombian VGO provided evidence that the zeolite Y present in the composite catalysts was accountable for their activity and for mitigating the deactivation effect caused by coke deposition. The CatY.0.20 catalyst had better performance in terms of conversion and selectivity to gasoline with improved quality as well as greater resistance to deactivation. The maximum gasoline selectivity observed with the CatY.0.20 catalyst was 50.9 wt%, whereas that with the CatY.0.00 catalyst was only 34.7 wt%. The treatment not only enhanced the gasoline cut performance, but also promoted a higher gasoline octane rating (CatY.0.20, RON = 88.2). At the same time, the CatY.0.00 catalyst was more selective to gases due to electronic confinement effects within the zeolite’s microporous channels.

The carbonaceous residues deposited in CatY.0.00 were predominantly aromatic in nature, as indicated by the FTIR band assigned to aromatic coke at 1580 cm^−1^. These residues primarily comprised four-membered aromatic rings (A4), constituting approximately 70% of its total composition. As expected, the textural and acidic properties of fresh versus coked catalysts were impacted by the deposition of coke on the catalyst surfaces. The CatY.0.00 catalyst was notably impacted by coke deposition, with its specific surface area experiencing losses of up to 70% and its acidity decreasing by up to 60%. Of the two catalysts studied, EcatY.0.20 presented the appropriate formulation to be used in refineries aimed at producing gasoline. These results underscore the significance of employing such laboratory reactors to investigate the performance of catalysts as an initial stage preceding industrial processes.

## Figures and Tables

**Table 1:** Textural and crystalline properties of zeolites and catalysts prepared from zeolites.

	Y-0.00-M	Y-0.20-M	CatY.0.00	CatY.0.20
BET specific surface area, *S_BET_* (m^2^/g)	693	436	362	355
Mesopore specific surface area, *S_meso_* (m^2^/g)	124	210	311	332
Total pore volume, *V_TP_* (cm^3^/g)	0.582	0.771	0.602	0.699
Micropore volume, *V_micro_* (cm^3^/g)	0.283	0.094	0.051	0.003
Mesopore volume, *V_meso_* (cm^3^/g)	0.299	0.677	0.551	0.669
Average mesopore diameter, ${\bar{d}}_{p}$ (Å)	64.2	84.3	66.7	99.7
Crystallinity (%)	93	29	13	5
Unit cell size (Å)	24.25	24.21	24.22	24.19

**Table 2:** The concentrations of Brönsted (B) and Lewis (L) acid sites (μmol Py/g) of zeolites Y-0.00-M and Y-0.20-M, and of catalysts CatY.0.00 and CatY.0.20 as determined by FTIR analysis.

	Desorption Temperature (°C)								
	150			300				450	
	B	L	B/L	B	L	B/L	B	L	B/L
Y-0.00-M	91	107	0.85	58	71	0.82	28	30	0.93
Y-0.20-M	93	135	0.70	61	84	0.73	29	48	0.61
CatY.0.00	26	35	0.81	9	13	0.80	16	18	0.81
CatY.0.20	35	45	0.79	20	27	0.79	13	15	0.77

**Table 3:** Distributions of products (selectivities, wt%) and composition of the gasoline in the conversion of VGO.

Conversion (wt%)	Higher		Lower	
	83.4	69.2	60.3	50.2
Selectivities (wt%)	CatY.0.20	CatY.0.00	CatY.0.20	CatY.0.00
S_DG_	21.5	28.5	13.8	20.5
S_LPG_	26.1	33.8	16.4	26.5
S_GASOLINE_	42.9	28.5	50.9	34.7
Gasoline composition (%)				
Paraffins	7.5	21.4	15.8	27.7
Olefins	30.4	32.1	22.5	25.1
Naphthenes	15.8	23.6	27.0	26.8
Aromatics	46.3	22.9	34.7	20.4
RON	88.2	79.8	86.4	81.1
S_COKE_ (wt%)	9.5	9.2	18.9	18.3

**Table 4:** Feedstock Properties.

°API	19.7
Aniline point (°C)	78.5
CCR (wt%)	0.43
Refractive index	1.49
Distillation curve (°C)	
Initial	272
10 vol.%	387
30 vol.%	420
50 vol.%	450
70 vol.%	487
95 vol.%	534
Final	582
SARA fractions (wt%)	
Saturated	47.4
Aromatic	50.0
Resin	2.10
Asphaltene	0.50
Nickel (ppm)	0.48
Vanadium (ppm)	0.97
Sodium (ppm)	0.83
Iron (ppm)	0.24
Sulfur (wt%)	1.12

## Data Availability

The data presented in this study are available in this article.
